# The Effect of Early Anticoagulation Therapy in Acute Pulmonary Embolism: A Retrospective Cohort Study in the Emergency Department

**DOI:** 10.7759/cureus.100980

**Published:** 2026-01-07

**Authors:** Amna Riaz, Noor Fatima, Qasim Ali, Natasha Kazi, Sunnia Khalid, Hafiz Usman Khalid Ranjha, Wesam Taher Almagharbeh, Lawson O Obazenu

**Affiliations:** 1 General Internal Medicine, Countess of Chester Hospital NHS Foundation Trust, Chester, GBR; 2 Biology, Raritan Valley Community College (RVCC), Branchburg, USA; 3 Nephrology, Renal Care Foundation, Faisalabad, PAK; 4 Emergency Medicine, Curative Care Company, Riyadh, SAU; 5 General Medicine, Ibne Sina Hospital, Multan, PAK; 6 Pulmonology, Fauji Foundation Hospital, Rawalpindi, PAK; 7 Medical and Surgical Nursing, University of Tabuk, Tabuk, SAU; 8 Medicine, University of Ibadan, Ibadan, NGA

**Keywords:** anticoagulation, emergency department, mortality, pulmonary embolism, thromboembolism

## Abstract

Background: Acute pulmonary embolism (PE) is a critical emergency condition that can lead to rapid hemodynamic compromise and death if not treated promptly.

Objective: To assess whether initiating anticoagulation within two hours of emergency department (ED) presentation improves in-hospital outcomes in patients with acute pulmonary embolism.

Methods: This retrospective cohort study was conducted at Fauji Foundation Hospital, Rawalpindi, from December 2024 to June 2025. A total of 345 adult patients diagnosed with acute PE between January 2020 and December 2024 were included. Patients were divided into two cohorts: early anticoagulation and delayed anticoagulation (initiated after four hours). Clinical data, including demographics, comorbidities, hemodynamic parameters, laboratory findings, imaging results, and treatment outcomes, were analyzed.

Results: Out of 345 patients, 192 (55.6%) received early and 153 (44.4%) delayed anticoagulation. The mean age was 54.8 ± 15.2 years, with 215 (62.3%) males. Mortality was significantly lower in the early group, 14 patients (7.3%), compared to 26 patients (17.0%) in the delayed group (p = 0.006). Early anticoagulation also reduced recurrent thromboembolism, occurring in seven patients (3.6%) versus 14 patients (9.2%) in the delayed group (p = 0.03), as well as ICU admissions-47 patients (24.5%) versus 59 patients (38.6%), respectively (p = 0.008). The mean hospital stay was significantly shorter in the early group (5.8 ± 2.9 days) than in the delayed group (8.4 ± 3.6 days, p < 0.001). No significant difference in major bleeding events was observed between groups (p = 0.48).

Conclusion: It is concluded that initiating anticoagulation within two hours of presentation significantly improves survival, decreases recurrence, and shortens hospital stay without increasing bleeding risk. Emergency departments should prioritize rapid diagnostic and treatment pathways to enable early anticoagulation, particularly in resource-limited settings.

## Introduction

Pulmonary embolism (PE) is a life-threatening complication of venous thromboembolism (VTE), a disorder that also involves deep vein thrombosis (DVT). It is a significant health issue in the world and is estimated to have an incidence of 60-70 cases per 100,000 people annually and it is thought to cause a significant percentage of cardiovascular mortality following myocardial infarction and stroke [1]. The pathophysiology of PE includes the blockage of the pulmonary arterial circulation by a thrombus, which is by far the most common to begin at the deep veins of the lower limbs. This blockage enhances pulmonary vascular resistance, causes right ventricular strain, failure, and eventually, the lack of gaseous exchange and hypoxia. In extreme cases, it can trigger sudden circulatory breakdown and fatality [2]. Most patients with hemodynamic distress present to the emergency department (ED) with massive or submassive PE despite the introduction of diagnostic imaging, including CT pulmonary angiography, and therapeutic modalities, have a case-fatality rate that is significant [3].

The clinical manifestation of PE is infamously interchangeable and non-specific, with mild dyspnea to syncope or cardiogenic shock. Therefore, late diagnosis is widespread and can deteriorate the prognosis. The key to the minimization of morbidity and mortality lies in the early identification and early therapeutic intervention [4]. Anticoagulation is the keystone of PE care, which is directed at preventing the extension of clots, repeated embolism, and pulmonary hypertension. Patient comorbidity, renal function, and hemodynamic condition inform the selection of an anticoagulant between unfractionated heparin, low-molecular-weight heparin (LMWH), fondaparinux, and direct oral anticoagulants (DOACs) [5]. Of these, unfractionated heparin is the most commonly used during the emergency environment because it has a rapid onset and reversibility. The numerous international guidelines, such as the ones of the European Society of Cardiology (ESC) and the American College of Chest Physicians (ACCP), underline that the anticoagulation therapy initiation should not be delayed in patients with high clinical suspicion of PE to get authoritative imaging [6]. Observational and interventional studies have continued to demonstrate with evidence that early anticoagulation enhances survival rates by reducing the development of the thrombus, as well as partially counteracting the right ventricular dysfunction [7]. Delayed therapy, in contrast, has been linked to a higher rate of short-term mortality and recurrent VTE, common to delayed therapy due to diagnostic uncertainty, inaccessibility of imaging, or ED congestion [8]. Nevertheless, even with the theoretically proven advantage, there is not enough practical evidence to measure the effect of the timing of starting anticoagulants and patient outcomes, especially in low- and middle-income nations where the resource scarcity might increase diagnostic-to-treatment periods. Research has revealed that the rate of mortality can be over 30% in untreated PE cases and reduce to less than 8% when anticoagulation is immediately instituted [9]. Early anticoagulation, as it is applicable to different studies, is described as early therapy that was started in either one to four hours after the presentations to the ED or clinical suspicion [10]. In tertiary hospitals with high volumes, logistical obstacles (e.g., the availability of diagnostic imaging, laboratory turnaround to D-dimer testing, and physician approval delays) can massively increase this window [11]. Further, comorbidities, such as renal dysfunction, hepatic dysfunction, or bleeding risk, can further make the decision on early intervention more complicated. Consequently, the clinical and operational significance of learning how early anticoagulation and late anticoagulation have a practical effect on patient outcomes in the emergency department is clinically and operationally relevant [12].

Objective

To assess whether initiating anticoagulation within two hours of ED presentation improves in-hospital outcomes in patients with acute pulmonary embolism.

## Materials and methods

This retrospective cohort study was conducted at Fauji Foundation Hospital, Rawalpindi, from December 2024 to June 2025 (Approval Number FFH/ERC/2024/2987). A total of 345 patients diagnosed with acute pulmonary embolism (PE) were included in the study. Non-probability consecutive sampling was used to include all eligible patients meeting the inclusion criteria during the study period.

Inclusion criteria

Patients of both sexes aged 18 years and above who presented to the emergency department and were diagnosed with acute pulmonary embolism based on clinical suspicion and confirmatory imaging (CT pulmonary angiography or ventilation-perfusion scan) were included. Patients were categorized as having massive, submassive, or low-risk PE according to hemodynamic parameters and imaging findings.

Exclusion criteria

Patients with chronic thromboembolic pulmonary hypertension, prior anticoagulation therapy for other indications before arrival to the emergency department, or incomplete medical records regarding the timing of anticoagulation initiation, were excluded.

Data collection procedure

Data were collected retrospectively from hospital medical records, electronic databases, and emergency department logs. Demographic details (age, gender), comorbidities (hypertension, diabetes, malignancy, and previous DVT), vital signs at presentation, diagnostic imaging reports, laboratory parameters (D-dimer, troponin, arterial blood gases), and time of anticoagulation initiation were recorded. The interval between hospital arrival and initiation of anticoagulant therapy was calculated and used to classify patients into two groups: Early anticoagulation group: Initiation of anticoagulation therapy within two hours of presentation to the emergency department. Delayed anticoagulation group: Initiation of therapy after two hours of presentation.

Anticoagulant regimens included unfractionated heparin, low-molecular-weight heparin, or direct oral anticoagulants, depending on clinical suitability and physician discretion. Patients requiring thrombolysis or surgical embolectomy were noted separately. The primary outcome was in-hospital mortality. Secondary outcomes included recurrent thromboembolic events, need for intensive care unit (ICU) admission, duration of hospital stay, and major bleeding complications as defined by the International Society on Thrombosis and Haemostasis (ISTH) criteria. Early anticoagulation therapy was compared with delayed initiation in relation to these outcomes.

Statistical analysis

Data were entered and analyzed using Statistical Package for the Social Sciences (SPSS) version 26.0 (IBM Corp., Armonk, NY, USA). Quantitative variables such as age, time to anticoagulation, and hospital stay were expressed as mean ± standard deviation (SD) or median with interquartile range (IQR), depending on data distribution. Categorical variables, such as gender, risk category, mortality, and recurrence, were presented as frequencies and percentages. The chi-square test or Fisher's exact test was applied for comparison of categorical variables, while the independent-samples t-test or Mann-Whitney U test was used for continuous variables as appropriate. A p-value ≤ 0.05 was considered statistically significant.

## Results

A total of 345 patients diagnosed with acute pulmonary embolism were included, with 192 (55.6%) receiving early anticoagulation and 153 (44.4%) treated later. The overall mean age was 54.8 ± 15.2 years, slightly younger in the early group (53.2 ± 14.6) compared to the delayed group (56.9 ± 15.8). Males predominated (62.3%), and hypertension (44.3%) and diabetes (31.6%) were the most frequent comorbidities. The proportion of patients with a history of deep vein thrombosis (DVT) was 16.8%, comparable between groups. Regarding PE severity, submassive cases were most common (58.8%), followed by massive (21.4%) and low-risk (19.8%) events. Patients receiving early anticoagulation exhibited higher mean systolic blood pressure (106 ± 22 mmHg) and oxygen saturation (93.0 ± 5.8%) than those with delayed therapy (98 ± 26 mmHg and 89.6 ± 7.2%, respectively), indicating better hemodynamic stability at presentation (Table 1).

**Table 1:** Baseline demographic and clinical characteristics of the study population (n = 345). DVT: deep vein thrombosis; PE: pulmonary embolism.

Variable	Total (n = 345)	Early anticoagulation (n = 192)	Delayed anticoagulation (n = 153)
Gender			
Male	215 (62.3%)	116 (60.4%)	99 (64.7%)
Female	130 (37.7%)	76 (39.6%)	54 (35.3%)
Age (years), mean ± SD	54.8 ± 15.2	53.2 ± 14.6	56.9 ± 15.8
Hypertension	153 (44.3%)	78 (40.6%)	75 (49.0%)
Diabetes mellitus	109 (31.6%)	57 (29.7%)	52 (34.0%)
History of DVT	58 (16.8%)	28 (14.6%)	30 (19.6%)
PE severity			
Massive	74 (21.4%)	35 (18.2%)	39 (25.5%)
Submassive	203 (58.8%)	117 (60.9%)	86 (56.2%)
Low risk	68 (19.8%)	40 (20.8%)	28 (18.3%)
Systolic BP (mmHg), mean ± SD	102 ± 24	106 ± 22	98 ± 26
Oxygen saturation (%), mean ± SD	91.5 ± 6.7	93.0 ± 5.8	89.6 ± 7.2
Troponin positive	88 (25.5%)	40 (20.8%)	48 (31.4%)
Time to anticoagulation (hours), mean ± SD	3.4 ± 2.9	1.4 ± 0.5	5.8 ± 2.7

Early anticoagulation was associated with a markedly lower in-hospital mortality rate (7.3%) compared to delayed initiation (17.0%, p = 0.006). Recurrent PE or DVT occurred less frequently in the early group (3.6% vs. 9.2%, p = 0.03), and ICU admissions were also reduced (24.5% vs. 38.6%, p = 0.008). The mean hospital stay was significantly shorter in patients receiving early therapy (5.8 ± 2.9 days) versus delayed treatment (8.4 ± 3.6 days, p < 0.001) (Table 2).

**Table 2:** Comparison of clinical outcomes between early and delayed anticoagulation groups. DVT: deep vein thrombosis; PE: pulmonary embolism.

Outcome	Early anticoagulation (n = 192)	Delayed anticoagulation (n = 153)	Test statistic	p-value
In-hospital mortality	14 (7.3%)	27 (17.0%)	χ² = 7.47	0.006
Recurrent PE/DVT	7 (3.6%)	14 (9.2%)	χ² = 4.69	0.03
ICU admission	47 (24.5%)	59 (38.6%)	χ² = 7.09	0.008
Mean hospital stay (days), mean ± SD	5.8 ± 2.9	8.4 ± 3.6	t = -7.03	<0.001
Major bleeding events	10 (5.2%)	8 (5.2%)	χ² = 0.00	0.48

Multivariate logistic regression identified massive PE (OR = 3.12, 95% CI: 1.54-6.31, p = 0.002), positive troponin (OR = 1.97, 95% CI: 1.01-3.85, p = 0.04), and delayed anticoagulation beyond two hours (OR = 2.45, 95% CI: 1.18-5.10, p = 0.016) as independent predictors of in-hospital mortality. Age above 65 years showed a non-significant trend toward higher mortality (OR = 1.84, p = 0.08) (Table 3).

**Table 3:** Multivariate logistic regression analysis for predictors of in-hospital mortality. PE: pulmonary embolism.

Variable	Adjusted odds ratio (OR)	95% confidence interval (CI)	p-value
Age >65 years	1.84	0.92-3.67	0.08
Massive PE	3.12	1.54-6.31	0.002
Positive troponin	1.97	1.01-3.85	0.04
Delayed anticoagulation (>2 hours)	2.45	1.18-5.10	0.016

Dyspnea (86.7%) and pleuritic chest pain (65.5%) were the most frequent presenting symptoms. Syncope was significantly more common in the delayed group (30.1%) than in the early group (17.2%, p = 0.006), reflecting greater hemodynamic compromise. Tachycardia (56.9% vs. 44.8%, p = 0.03) and hypoxia (37.9% vs. 22.4%, p = 0.002) were also more prevalent among those with delayed anticoagulation (Table 4).

**Table 4:** Distribution of presenting symptoms and risk factors among study participants (n = 345).

Clinical feature	Total (n = 345)	Early anticoagulation (n = 192)	Delayed anticoagulation (n = 153)	Test statistic	p-value
Dyspnea	299 (86.7%)	169 (88.0%)	130 (85.0%)	χ² = 0.63	0.43
Pleuritic chest pain	226 (65.5%)	127 (66.1%)	99 (64.7%)	χ² = 0.07	0.79
Cough	118 (34.2%)	61 (31.8%)	57 (37.3%)	χ² = 1.12	0.29
Hemoptysis	54 (15.7%)	27 (14.1%)	27 (17.6%)	χ² = 0.78	0.38
Syncope	79 (22.9%)	33 (17.2%)	46 (30.1%)	χ² = 7.62	0.006
Tachycardia (>100 bpm)	173 (50.1%)	86 (44.8%)	87 (56.9%)	χ² = 4.68	0.03
Hypoxia (SpO₂ < 90%)	101 (29.3%)	43 (22.4%)	58 (37.9%)	χ² = 9.59	0.002
Recent surgery (<1 month)	47 (13.6%)	28 (14.6%)	19 (12.4%)	χ² = 0.34	0.56
Active malignancy	31 (9.0%)	14 (7.3%)	17 (11.1%)	χ² = 1.44	0.23
Prolonged immobilization (>3 days)	72 (20.9%)	39 (20.3%)	33 (21.6%)	χ² = 0.08	0.77

Patients with delayed anticoagulation had higher mean D-dimer levels (2240 ± 755 ng/mL vs. 2080 ± 695 ng/mL, p = 0.04) and lower arterial pO₂ (64.2 ± 13.6 vs. 69.5 ± 11.4 mmHg, p = 0.002), suggesting more severe hypoxemia. Elevated BNP (>100 pg/mL) and right ventricular dysfunction on echocardiography were more frequent in the delayed group (29.4% and 33.3%, respectively) compared to the early group (16.1% and 21.9%), both statistically significant. Segmental or multilobar thrombus involvement on CT pulmonary angiography was also more common among patients with delayed therapy (64.7% vs. 53.1%, p = 0.04) (Table 5).

**Table 5:** Laboratory and imaging findings in patients with acute pulmonary embolism. BNP: B-type natriuretic peptide; PE: pulmonary embolism.

Parameter	Total (n = 345)	Early anticoagulation (n = 192)	Delayed anticoagulation (n = 153)	Test statistic	p-value
D-dimer (ng/mL), mean ± SD	2150 ± 720	2080 ± 695	2240 ± 755	t = -2.05	0.04
Arterial pO₂ (mmHg), mean ± SD	67.2 ± 12.9	69.5 ± 11.4	64.2 ± 13.6	t = 3.12	0.002
Arterial pCO₂ (mmHg), mean ± SD	33.8 ± 5.6	33.1 ± 5.2	34.8 ± 6.1	t = -2.17	0.03
BNP elevated (>100 pg/mL)	76 (22.0%)	31 (16.1%)	45 (29.4%)	χ² = 8.31	0.004
Right ventricular dysfunction on echo	93 (27.0%)	42 (21.9%)	51 (33.3%)	χ² = 5.39	0.02
CT pulmonary angiography confirmed PE	321 (93.0%)	178 (92.7%)	143 (93.5%)	χ² = 0.08	0.77
Segmental/multilobar involvement	201 (58.2%)	102 (53.1%)	99 (64.7%)	χ² = 4.20	0.04
Pulmonary infarction	63 (18.3%)	29 (15.1%)	34 (22.2%)	χ² = 2.90	0.09

## Discussion

This retrospective cohort study shows that initiating anticoagulation therapy early significantly reduces in-hospital mortality, thromboembolic recurrence, and length of stay in patients with acute pulmonary embolism (PE) without increasing major bleeding risk. In accordance with established international recommendations, these findings strongly support the principle of prompt anticoagulation as a lifesaving measure in PE management. Our study's overall mortality rate of 11.9% is consistent with global estimates that range from 8% to 15% for PE-related in-hospital mortality. However, the clinical significance of time-sensitive therapy is emphasized by the significant difference in mortality rates between the early (7.3%) and delayed (17.0%) anticoagulation groups. This outcome reinforces the notion that the first few hours following presentation are critical for hemodynamic stabilization and preventing clot propagation. Similar outcomes were observed in the Sharifi et al. [13] study, which found that when compared to delayed treatment, immediate initiation of heparin therapy significantly improved right ventricular function and reduced adverse events. In a similar vein, the 2019 guidelines issued by the European Society of Cardiology (ESC) recommend initiating anticoagulation as soon as possible in all patients who have been diagnosed with PE or have a high level of suspicion for it, particularly in situations where diagnostic delays are anticipated. Our findings also show that early anticoagulation was associated with shorter hospital stays and fewer admissions to the intensive care unit (ICU), suggesting that timely therapy not only increases survival rates but also improves resource utilization and reduces costs. These findings mirror the outcomes reported by Kearon and colleagues, who demonstrated that early therapeutic anticoagulation is associated with faster clinical recovery and decreased hospitalization duration [14]. In addition, early treatment may be able to break the vicious cycle of hypoxia and strain on the right ventricle, preventing clinical deterioration and the need for more advanced treatments like thrombolysis or mechanical ventilation. Patients with comorbidities like renal impairment or diagnostic uncertainty were more likely to experience delayed anticoagulation in our study, which frequently required confirmatory imaging and laboratory clearance. These practical obstacles are prevalent in emergency settings, particularly in hospitals with limited resources, where diagnostic facilities like CT pulmonary angiography (CTPA) may not be readily available at all times [15]. Similar operational constraints have been reported in low- and middle-income countries, where delays in imaging and consultation contribute to suboptimal outcomes in PE management. According to ACCP and ESC guidelines, these findings highlight the need for standardized protocols that permit empirical anticoagulation in patients with high clinical suspicion pending imaging confirmation. The study further identified massive PE and delayed anticoagulation as independent predictors of in-hospital mortality. Because massive PE is characterized by hemodynamic instability, systemic hypotension, and right ventricular failure, all of which increase the risk of death, this observation is biologically plausible. Importantly, our logistic regression model demonstrated that delayed anticoagulation more than doubled the odds of death, even after adjusting for confounders such as age and comorbidities [16]. This highlights that the timing of therapy initiation independently influences outcomes, irrespective of baseline disease severity. Interestingly, the incidence of major bleeding was comparable between the two groups, indicating that early anticoagulation did not confer a higher bleeding risk. This finding aligns with data from the Pulmonary Embolism International Thrombolysis (PEITHO) and Hokusai-VTE trials, where early anticoagulation, particularly with LMWH, demonstrated a favorable safety profile, even among elderly and comorbid patients. The non-significant difference in bleeding rates suggests that concerns about early therapy leading to hemorrhagic complications are often overstated, provided dosing and monitoring are carefully implemented [17].

Our study's predominance of male patients and mean age of patients (54.8 years) are in line with previous epidemiological reports from Middle Eastern and South Asian populations. The high prevalence of hypertension and diabetes among PE patients may reflect the growing burden of metabolic syndrome in developing countries, which contributes to endothelial dysfunction and prothrombotic states [18]. The multifactorial nature of venous thromboembolism in emergency admissions is also emphasized by the prominent risk factors of immobility, recent surgery, and active cancer. The clinical implications of our findings are significant. First, when diagnostic delays are anticipated, emergency physicians should prioritize early assessment and empiric anticoagulation in patients with moderate to high Wells or Geneva scores, even before confirmatory imaging. Second, hospitals need to come up with simplified routes that will allow for quicker access to imaging, early heparin administration, and laboratory processing. Thirdly, in unstable patients, integrating bedside echocardiography for risk stratification can speed up therapeutic decision-making. In actual emergency situations, putting such measures into action could significantly increase survival rates and decrease the number of complications [19].

Limitations

This study had several limitations that should be acknowledged. Its retrospective design depended heavily on the accuracy and completeness of existing medical records, which introduced the possibility of information bias. The single-center nature of the study may limit the applicability of the findings to other institutions with different patient populations, clinical practices, and resource availability. The study did not stratify outcomes according to specific anticoagulant regimens, which may have influenced variations in treatment response or bleeding tendencies. Long-term outcomes were also not evaluated, preventing assessment of recurrence rates or the development of chronic complications after discharge. Despite these limitations, the study’s relatively large sample size and real-world emergency department setting provided meaningful insights and supported the external validity of the results.

## Conclusions

Early initiation of anticoagulation therapy in acute pulmonary embolism significantly improves clinical outcomes, reduces mortality, and shortens hospital stay without increasing bleeding risk. Delays in initiating therapy remain a modifiable factor contributing to poor prognosis. Institutions should adopt fast-track diagnostic and treatment protocols to ensure that anticoagulation is administered within the first few hours of presentation, particularly in resource-limited settings where diagnostic delays are frequent.

## References

[1] Smith SB, Geske JB, Maguire JM, Zane NA, Carter RE, Morgenthaler TI (2010). Early anticoagulation is associated with reduced mortality for acute pulmonary embolism. Chest.

[2] Hayashi H, Tsuji A, Kotoku A (2024). Effect of direct oral anticoagulant therapy on pulmonary artery clot dissolution in intermediate high-risk pulmonary thromboembolism. Thromb J.

[3] Prucnal CK, Jansson PS, Deadmon E, Rosovsky RP, Zheng H, Kabrhel C (2020). Analysis of partial thromboplastin times in patients with pulmonary embolism during the first 48 hours of anticoagulation with unfractionated heparin. Acad Emerg Med.

[4] Burge AJ, Freeman KD, Klapper PJ, Haramati LB (2008). Increased diagnosis of pulmonary embolism without a corresponding decline in mortality during the CT era. Clin Radiol.

[5] Zhu E, Yuriditsky E, Raco V (2024). Anti-factor Xa as the preferred assay to monitor heparin for the treatment of pulmonary embolism. Int J Lab Hematol.

[6] Jiménez Castro D, Sueiro A, Díaz G (2007). Prognostic significance of delays in diagnosis of pulmonary embolism. Thromb Res.

[7] Raskob GE, Van Es N, Verhamme P (2018). Edoxaban for the treatment of cancer-associated venous thromboembolism. N Engl J Med.

[8] Sanchez O, Trinquart L, Colombet I, Durieux P, Huisman MV, Chatellier G, Meyer G (2008). Prognostic value of right ventricular dysfunction in patients with haemodynamically stable pulmonary embolism: a systematic review. Eur Heart J.

[9] Kucher N, Goldhaber SZ (2003). Cardiac biomarkers for risk stratification of patients with acute pulmonary embolism. Circulation.

[10] Witt DM, Nieuwlaat R, Clark NP (2018). American Society of Hematology 2018 guidelines for management of venous thromboembolism: optimal management of anticoagulation therapy. Blood Adv.

[11] Robertson L, Jones LE (2017). Fixed dose subcutaneous low molecular weight heparins versus adjusted dose unfractionated heparin for the initial treatment of venous thromboembolism. Cochrane Database Syst Rev.

[12] Stubblefield WB, Helderman R, Strokes N, Greineder CF, Barnes GD, Vinson DR, Westafer LM (2025). Factors in initial anticoagulation choice in hospitalized patients with pulmonary embolism. JAMA Netw Open.

[13] Sharifi M, Bay C, Skrocki L, Rahimi F, Mehdipour M (2013). Moderate pulmonary embolism treated with thrombolysis (from the "MOPETT" Trial). Am J Cardiol.

[14] Nutescu EA, Burnett A, Fanikos J, Spinler S, Wittkowsky A (2016). Pharmacology of anticoagulants used in the treatment of venous thromboembolism. J Thromb Thrombolysis.

[15] Konstantinides SV, Meyer G, Becattini C (2020). 2019 ESC Guidelines for the diagnosis and management of acute pulmonary embolism developed in collaboration with the European Respiratory Society (ERS). Eur Heart J.

[16] Yamashita Y, Morimoto T, Amano H (2018). Anticoagulation therapy for venous thromboembolism in the real world-from the COMMAND VTE Registry. Circ J.

[17] Leentjens J, Peters M, Esselink AC, Smulders Y, Kramers C (2017). Initial anticoagulation in patients with pulmonary embolism: thrombolysis, unfractionated heparin, LMWH, fondaparinux, or DOACs?. Br J Clin Pharmacol.

[18] Raharjo PP, Wiyono AV, Panjaitan U, Putra IC, Hermanto K, Dewi TI, Pramudyo M (2025). Life-threatening recurrent pulmonary embolism following anticoagulation withdrawal: a case report emphasising the management dilemma in resource-constrained settings. Int J Emerg Med.

[19] Chen D, Bhaskar SM (2025). Pulmonary embolism in acute ischaemic stroke: evolving evidence, diagnostic challenges, and a novel thromboinflammatory axis hypothesis. Int J Mol Sci.

